# Ureteral Avulsion during Semirigid Ureteroscopy: A Single-Centre Experience

**DOI:** 10.1155/2020/3198689

**Published:** 2020-10-19

**Authors:** A. R. Bhaskarapprakash, Leelakrishna Karri, P. Velmurugan, S. Venkatramanan, K. Natarajan

**Affiliations:** Department of Urology, Sri Ramachandra Institute of Higher Education and Research, Chennai, India

## Abstract

**Aims:**

The aim of the study is to present our experience with the management of ureteral avulsions following semirigid ureteroscopy for ureteral stones. This is one of the largest series reported so far.

**Methods and Materials:**

It is a retrospective and observational study done at Sri Ramachandra Institute of Higher Education and Research over the last 18 years.

**Results:**

There were seven cases of ureteral avulsion following semirigid ureteroscopy. All patients were males with a mean age of 35.7 years. All had impacted stones, with proximal ureteric location in 6 patients and distal ureteric location in 1 patient. Five cases had two-point avulsions with loss of entire ureter. Two cases had one-point avulsion: one distal ureteric and the other mid-ureteric. Of the five cases with whole length ureteral avulsion, four were managed by classical ileal replacement of ureter and the the fifth case was managed by ileal replacement of ureter by the Yang–Monti technique. Of the two cases with one-point avulsion, one was managed by uretero-neocystostomy and the other by uretero-ureterostomy. All the patients had successful outcome.

**Conclusions:**

Even though rare, ureteral avulsion can potentially happen especially when dealing with impacted ureteric stones. Being conscious of the possible occurrence of this serious complication during any difficult ureteroscopy and exercising utmost care during the procedure are important preventive measures. However, this catastrophe can be successfully managed by either immediate definitive repair or in a staged manner.

## 1. Introduction 

Semirigid ureteroscopy is widely used as the therapeutic and diagnostic procedure for various renal and ureteric conditions, with management of ureteric stones being one of the most common indications [1]. It is generally an easy, simple, and efficacious procedure which can be done on a daycare basis. With advances in the technology of scopes and accessories, the use of semirigid ureteroscopy has been increasing steadily and it is now being used for even large proximal ureteric stones [2]. Even though it is generally a safe procedure, it can be associated with an overall complication rate of more than 50% [3]. Fortunately, most of these complications are insignificant or minor and are amenable to conservative measures without affecting the outcome of the procedure. Ureteral avulsion is a rare but a disastrous complication of ureteroscopy, and it converts a simple minimally invasive procedure into a major and complex open surgical exercise with potential for significant sequelae. We present our experience with the management of ureteral avulsions following semirigid ureteroscopy for ureteral stones.

## 2. Subjects and Methods

It is a retrospective and observational study done at Sri Ramachandra Institute of Higher Education and Research over the last 18 years. The ureteral avulsions following semirigid ureteroscopy for ureteral stones that occurred in-house in our institute as well as the ureteral avulsions that were referred to our institute from other centres were included in the study.

The clinical summary of the 7 cases of ureteric avulsion is mentioned in Table 1.

## 3. Case Reports

### 3.1. Patient 1

**Figure 1:**
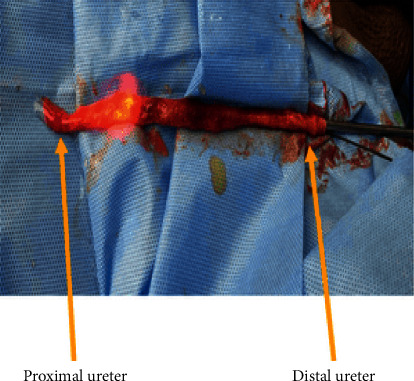
Complete (two-point) ureteral avulsions following semirigid ureteroscopy.

**Figure 2:**
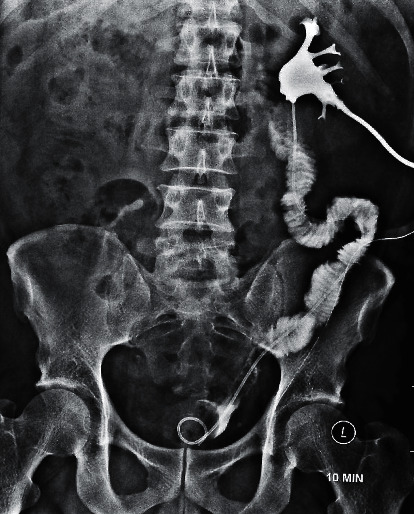
Nephrostogram after classic ileal replacement with adequate drainage.

A 31-year-old male presented with colicky left loin to groin pain of 2 months' duration. There was an 11 mm left proximal ureteric calculus with moderate hydroureteronephrosis on plain CT KUB, and serum creatinine was normal. The ureteroscopy was done using a 6/7.5 Fr semirigid ureterorenoscope under spinal anaesthesia. The ureter up to the level of the stone was friendly, but the stone was impacted and the ureter at that level was tight. The stone was fragmented with lithoclast. But during the attempt to remove the stone fragments with a stone basket, the ureter snapped resulting in scabbard avulsion of nearly the entire length of the ureter (Figure 1). As the patient was stable, immediate laparotomy and classical ileal replacement of the entire ureter was done under general anaesthesia. The ileal ureter was splinted by a DJ stent and protected by a nephrostomy tube. The postoperative nephrostogram revealed a well-sitting ileal ureter (Figure 2).

### 3.2. Patient 2

A 39-year-old male with left ureteric colic of 2 weeks duration due to an impacted 6.5 mm left proximal ureteric calculus with moderate hydroureteronephrosis. The ureteroscopy was done with a 6/7.5 Fr ureterorenoscope. The entry into the ureter up to the level of stone was fairly easy. The stone was impacted, and the site of impaction was friable. During the process of stone fragmentation and disengaging the fragments from the impacted site, the ureter snapped at the proximal end and the entire ureter avulsed. This patient was again managed successfully by immediate laparotomy and classical ileal replacement of ureter.

### 3.3. Patient 3

A 25-year-old male with right ureteric colic of 3-week duration had an impacted 1.2 cm calculus at the level of L-4 in the right ureter. Ureteroscopy was done with an 8/9.8 Fr ureterorenoscope. Preliminary dilatation of the ureteric orifice was not required to enter. A relatively large stone fragment was trapped in the stone basket, but the process of withdrawal of the ureteroscope with dormia with its entrapped stone was difficult and suddenly the ureter suffered one-point avulsion at the stone impaction site. The procedure was stopped, and no further attempt was made to extricate the instruments from the ureter and was left in situ. Immediate laparotomy was made, and under vision, the stone fragment was dislodged from the basket and then instruments were delicately disengaged from the lower ureteral stump and removed without causing any further damage to the ureteral stump. The edges of the upper and lower ureteral stumps were freshened, and end-to-end uretero-ureteral anastomosis was done over a double *J* stent. The retrograde ureterogram done at the time of stent removal at 6 weeks showed a normal ureteral calibre.

### 3.4. Patient 4

A 28-year-old male diagnosed with impacted left distal ureteric calculus of 1.2 cm size just proximal to the vesico-ureteric junction underwent ureteroscopy with an 8/9.8 Fr semirigid ureterorenoscope. As the ureteric orifice was tight, it was dilated with a balloon dilator. But the subsequent attempt at entry with the ureteroscope caused injury to the intramural and juxta vesical ureter, leading to avulsion of the ureter from the bladder. On withdrawal of the ureteroscope, a 2 cm length of a distal ureter was seen to be draped over the scope. Immediate laparotomy and ureteric reimplantation were done by the same technique as uretero-vesical anastomosis of a transplant ureter.

### 3.5. Patient 5

**Figure 3:**
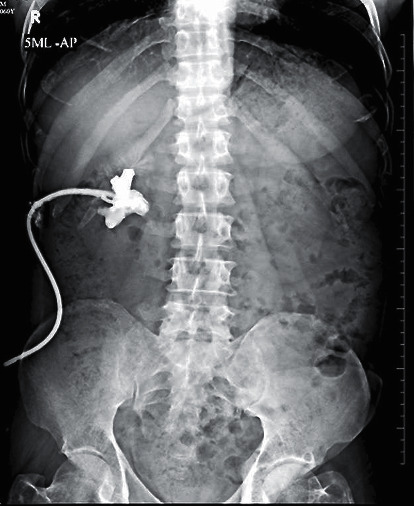
Complete (two-point) ureteral avulsions with percutaneous nephrostomy (PCN) in situ. Nephrostogram showing blind-ending pelviureteric junction (PUJ).

**Figure 4:**
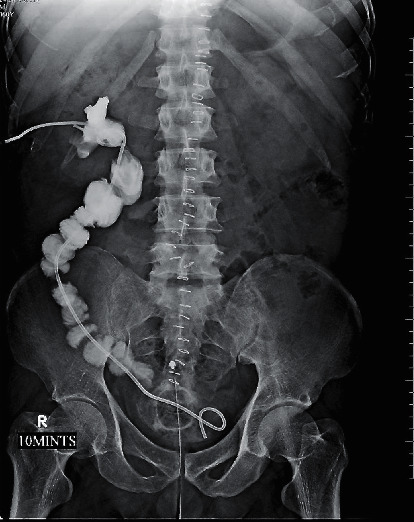
Classic ileal replacement done after 3 weeks. Nephrostogram with adequate drainage.

A 36-year-old male with an impacted 1.2 cm right proximal ureteric calculus initially underwent ureteroscopy with a 4.5/6 Fr semirigid ureterorenoscope in a hospital in another city. There was a complete avulsion of the ureter during the procedure. After managing the emergency with ultrasound-guided nephrostomy, the patient was referred to our centre 10 days later (Figure 3). Classical ileal replacement of the entire ureter was done at our centre at three weeks (Figure 4).

### 3.6. Patient 6

A 60-year-old male initially underwent ureteroscopy for 1 cm right proximal ureteric calculus with a 6/7.5 Fr ureterorenoscope in another state. During the procedure, there was ureteral avulsion which was managed by immediate exploratory laparotomy. As the ureter could not be found, a nephrostomy was done. He presented to our centre 5 months later with a nephrostomy tube in situ. On exploration at our centre, there was no trace of the right ureter. The condition was managed by classical ileal replacement of ureter.

### 3.7. Patient 7

**Figure 5:**
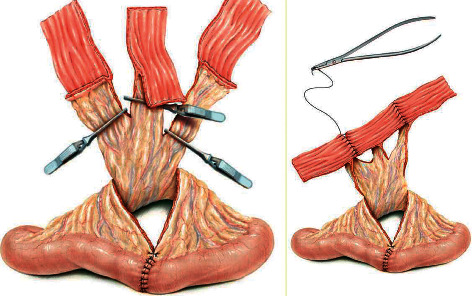
Yang–Monti technique (from M. A. Ghoneim and B. Ali-El-Dein, “Surgical atlas replacing the ureter by an ileal tube, using the Yang–Monti procedure. Illustrations by Stephan Spitzer,” *BJU International*, 462–463, 2005).

A 31-year-old male underwent ureteroscopy with a 6/7.5 Fr semirigid ureterorenoscope for a 1.3 cm left proximal ureteric calculus in another state. On arrival to our hospital 24 hours later, the emergency was initially managed with percutaneous nephrostomy. Subsequently, 3 weeks later ileal replacement of the ureter was done using the Yang–Monti method wherein 4 ileal segments had to be used to bridge the gap (Figure 5).

## 4. Results

There were 7 cases of ureteral avulsion. Four of them happened in-house in our institute and 3 were referred from other centres. All the cases of avulsion were healthy males with age ranging from 25 years to 60 years (mean 35.7 years). The avulsion involved the left side in 4 and right in 3 cases. All the patients had impacted stones with the proximal ureteric location in 6 and distal ureteric location in 1. In 6 patients, the stones were large (10–13 mm). The size of the semirigid ureteroscopes used ranged from 8/9.8F to 4.5/6F. Five cases had two-point avulsions with loss of entire ureter. Two cases had one-point avulsion: one distal ureteric and the other mid-ureteric. Two avulsions were caused by senior consultants, 1 by a junior consultant, 1 by a trainee, and 3 by urologists in other centres.

One distal ureteric avulsion was repaired immediately by uretero-neocystostomy with psoas hitch. In the patient with one-point mid-ureteric avulsion, the ureteroscope with an open stone basket was stuck at the injured site and it was difficult to disengage them. Instead of any further forceful maneuver, the instruments were left in situ and immediate exploratory laparotomy was done to free the instruments from the fragile distal ureteric stump. It was followed by uretero-ureterostomy.

Two patients with in-house two-point avulsion were managed with immediate laparotomy and classical ileal replacement of ureter.

Three referred cases with two-point avulsion were initially managed with percutaneous nephrostomy (PCN). Subsequent definitive repair was done after a variable period of 3 weeks to 5 months. In two of these cases, the classical ileal replacement of the entire ureter using an untailored distal ileal segment was done. In the third patient, ileal replacement of the entire ureter was done by the Yang–Monti technique using 4 segments of the ileum.

All the patients have had a satisfactory outcome with no significant further complications. Two patients were lost to follow-up after four years and five years, respectively. The other five patients continue to be in the follow-up with well-functioning renal units.

## 5. Discussion

Ureteric avulsion following ureteroscopy is a rare but a grave and dreaded complication. The incidence of ureteral avulsion reported in the earlier series was about 0.5% [4]. The later series seemed to suggest a fall in the incidence to about 0.1% or less probably due to technological advances [5–7]. However, some of the recent studies have again quoted a higher ureteral avulsion rate of more than 0.4% [8]. In fact, the study by Ercan Ogreden et al. published in 2016 reported an avulsion rate of 0.86%, which is one of the highest in the literature so far [3]. Ureteric avulsion incidence in our series is 0.05% (4 ureteric avulsions had happened in our institute out of 8640 total ureteroscopy procedures). Hence, ureteral avulsion, even though rare, is an ever-present threat. Avulsion is frequently the result of extraction of a large calculus through narrow ureter or due to blind basket extraction [9]. Even in the absence of such surgical misadventures, avulsion can still happen unpredictably. The ureters which are unhealthy or weak because of large impacted stones or previous ureteroscopic manipulations are particularly at risk. [10]. The upper third of the ureter is more prone to avulsion because of relatively less muscular tissue support.

Despite consisting of only seven cases of ureteral avulsion, the present study is one of the largest studies reported. The study by Ercan Ogreden et al. also had 7 cases of ureteral avulsion, but the avulsions were managed in three different centres [3]. A small number of cases make meaningful statistical analysis difficult. Some observations can be made regarding the cases and their management. All our patients with avulsion were healthy and relatively young (mean age of 35.7 years), and none of them had any significant adverse general health condition that could have made ureteroscopic procedure risky. All seven patients were males. This raises the question whether male gender is a risk factor for avulsion and whether female ureter is more resilient due to different hormonal milieu or different anatomy from that of males. As far as the gender correlation with complications of ureteroscopy is concerned, the previous studies have been of the view that overall complications were similar in both genders [7, 11, 12]. One study reported that only grade 1 complications, mainly mild haematuria, were more common in males [3]. But in the view of rarity of ureteral avulsion, the avulsion-specific gender correlation may be difficult to establish statistically.

The size of the ureteroscope has been repeatedly shown to be a positive risk factor for overall complications [3, 5, 13–15]. It is assumed that the same applies to avulsion as well. But this should not lead to a false assurance that small scopes are always safe with regard to avulsion. In our study, avulsion was seen even in a patient in whom a 4.5–6 F ureteroscope was used. The intrinsic fragility of the unhealthy ureter, especially the one with a large impacted stone, could be a more important factor for avulsion than the scope size in some cases that the ureter snaps unpredictably even with minor manipulations.

The following features help in the recognition of impending ureteric injury:

- Preoperatively:
- Presence of a large impacted proximal ureteric calculus especially in a male (present study)
- History of prior ureteral surgeries or procedures which could have resulted in ureteral fibrosis or loss of ureteral elasticity [16]

(ii) Intraoperatively:
(1) Difficulty in manipulation of an impacted ureteral stone
(2) Forceful basketing [17, 18]
(3) Attempted removal of a stone fragment too large for the ureter to accommodate [10]
(4) Resistance or a gripping sensation of the ureter, a little force is required to advance or withdraw the scope [19]

In the presence of the above factors, the disintegration of a stone by dusting technique and avoiding the use of a basket reduce the risk of ureter injury [19]. In case of difficulty or doubt, stenting and staging the procedure will be a safe option.

Another important factor with respect to avulsion is the level of experience of the surgeon doing ureteroscopy. The general dogma which is true for any surgery is that the more experienced the surgeon, the lesser the complication. But again because of its rarity, it is difficult to prove or disprove this correlation statistically for ureteral avulsion. Ercan Ogreden et al. had reported that ureteral avulsion happened in the hands of both experienced and less-experienced surgeons [3]. We also observed similar findings in our study where two avulsions were caused by senior consultants, one by a junior consultant, one by a urology resident, and three by practicing urologists elsewhere with varying levels of experience. The ureteral avulsion is rare and unpredictable and when it happens, it is catastrophic. Hence, even a very experienced surgeon should not lose one's guard and should take utmost precaution in every ureteroscopy to prevent this complication. The important components of precaution are constant awareness of the possible occurrence of this complication in every case, avoidance of forceful maneuvers and staging the procedure, or opting for alternative treatment modality in case of difficulty. It is very important not to forcibly try to pull out the ureteroscope or working instrument if it gets stuck in the ureter. It is better to leave the instrument in situ and do laparotomy to disengage the instrument gently, thereby preventing or limiting the ureteral damage. This was done in one of our cases with mid-ureteral avulsion with ureteroscope and basket stuck in the ureter. Laparotomy and disengagement of the instruments prevented one-point avulsion from progressing to two-point avulsion with loss of distal ureteral segment. Thereby, the ureteral injury could be managed with end-to-end ureteral anastomosis rather than a more extensive ureteroneocystostomy with Boari flap.

The repair of ureteral avulsion is preferably done immediately because delaying it may lead to loss of ureteral length due to ensuing fibrosis. Moreover, the patient does not have to undergo the trouble of waiting for weeks on a nephrostomy tube for the definitive repair. However, if the conditions are not favourable based upon the available surgical expertise or operation theatre facility or patient's general condition, the initial management will consist of only percutaneous nephrostomy. But ultimately, whether immediate or delayed, the outcomes in our series have been uniformly good. The suggested time frame for delayed repair is about 4 to 8 weeks.

Various definitive management options have been described based upon the type of injury whether one-point or two-point injury, site of injury, and the extent of ureteral loss as well as the condition of the renal unit and that of the patient. The options are ureteropyelostomy [20], ureteropyelostomy with omental wrapping [21], end-to-end ureteral anastomosis [3], uretero-vesical anastomosis with psoas hitch or Boari flap [3, 10], transuretero-ureterostomy [22], appendix interposition of the ureter [23, 24], simple repositioning of the completely avulsed ureter [6], pyeloureterostomy plus greater omentum investment outside the avulsed ureter and uretero-vesical anastomosis [25, 26], extended spiral bladder flap [20], ileal replacement of ureter [27], and finally nephrectomy [9, 10, 28] and auto renal transplantation [29].^.^

Ileal conduit is a simple and time-tested procedure. Even though nephrectomy with autotransplantation is also an option, there is paucity of large studies comparing the two procedures head-to-head. Nephrectomy with autotransplantation is beset with certain disadvantages: it is technically a more demanding procedure, and the expertise of which may not be easily available. Any problem with renal cooling and/or vascular anastomosis can cause irreparable damage to the kidney. Multiple vessels, if present, may complicate the procedure and its outcome. In cases with complete ureteral loss, which happens often, even if renal auto transplantation is attempted, the gap between the renal pelvis and the bladder may still have to be bridged using an intestinal segment or some other mechanism which may add to the complexity of the procedure.

For complete ureteral avulsion, the classical ileal replacement of ureter is a very reliable salvage option especially in the situation of immediate repair. Four of our 5 complete ureteral avulsion cases underwent classical ileal replacement of ureter without any significant intraoperative or postoperative complications. Despite its high success rate, ileal ureter has its own intrinsic risks in the form of mucus secretion and mucus plug formation, associated bowel complications (ileus, bowel obstruction) and anastomotic narrowing and obstructive uropathy, recurrent pyelonephritis, renal calculi, and dyselectrolytemia. In view of all this, the procedure described by Gao et al. and Tang et al. for complete ureteral avulsion (repositioning of the avulsed ureter by stented uretero-pyelostomy and uretero-vesical anastomosis, followed by omental wrapping of the ureter) appears very attractive [25, 26]. If its efficacy is validated by further studies, it may become the preferred option. One of our patients with complete ureteral avulsion underwent a staged definitive repair 3 weeks after the injury by ileal replacement of ureter by the Yang–Monti technique using 4 ileal segments. The outcome was satisfactory. The use of shorter length of ileum reduces the bowel-related complications as compared to classical ileal replacement of the ureter. However, because of the longer operative time and technical complexity, it may be more appropriate in the situation of staged repair rather than immediate repair.

## 6. Conclusion

Two-point ureteral avulsion was commoner than one-point avulsion. Risk factors for ureteral avulsions were male gender and relatively large impacted proximal ureteral stones. Small size of the scope and long experience of the surgeon are not necessarily safeguarding against ureteral avulsion. Prevention involves constant awareness of its possible occurrence during any ureteroscopy, avoidance of forceful endoscopic maneuver, and staging the procedure in case of difficulty. The classical ileal replacement of ureter is a reliable salvage option in the acute situation. The Yang–Monti technique of ileal replacement of ureter is also a viable option in a stable patient who has been managed initially with percutaneous nephrostomy for few weeks.

## Figures and Tables

**Table 1:** Clinical summary of 7 patients and their management.

Case no.	Age/gender	Indication	Size of scope used	Centre where avulsion occurred	Operating surgeon	Type of avulsion	Type and time of intervention
1	31/M	Left proximal ureteric calculus (1.1 cm, impacted)	6/7.5 Fr	In-house	Junior consultant	Two-point avulsion with complete loss of ureter (Figure 1)	Immediate repair; classical ileal replacement of ureter. Anastomosis was confirmed by nephrostogram (Figure 2)
2	39/M	Left proximal ureteric calculus (6.5 mm, impacted)	6/7.5 Fr	In-house	Senior consultant	Two-point avulsion with complete loss of ureter	Immediate repair; classical ileal replacement of ureter
3	25/M	Right proximal ureteric calculus (1.2 cm, impacted)	8/9.8 Fr	In-house	Resident	One-point mid-ureteric avulsion. Ureteroscope and stone basket protruding out of the friable distal ureteric stump and not disengageable	Immediate repair; laparotomy, disengagement of ureteroscope, and basket from the distal ureteric stump followed by uretero-ureterostomy
4	28/M	Left distal ureteric calculus (1.2 cm, impacted)	8/9.8 Fr	In-house	Senior consultant	One-point distal ureteric avulsion	Immediate repair; Uretero-neocystostomy with psoas hitch
5	36/M	Right proximal ureteric calculus (1.2 cm, impacted)	4.5 Fr	Referred from another centre	Practising urologist	Two-point avulsion with complete loss of ureter. With antegrade study—showing blind-ending pelviureteric junction (Figure 3)	Immediate PCN; classical ileal replacement of ureter after 3 weeks. Anastomosis was confirmed by nephrostogram (Figure 4)
6	60/M	Right proximal ureteric calculus (1 cm, impacted)	6/7.5 Fr	Referred from another centre	Practising urologist	Two-point avulsion with complete loss of ureter	Immediate PCN; classical ileal replacement of ureter after 5 months
7	31/M	Left proximal ureteric calculus (1.3 cm impacted)	6/7.5 Fr	Referred from another centre	Practising urologist	Two-point avulsion with complete loss of ureter	Immediate PCN tube insertion and ileal replacement of ureter (Yang–Monti) after 3 weeks (Figure 5)

## Data Availability

No data were used to support this study.
